# A Brighton Infirmary for Indian Troops

**Published:** 1914-12-05

**Authors:** 


### A BRIGHTON INFIRMARY FOR INDIAN TROOPS.

The war has made, and is still making, many changes in the Poor-Law service. Beds have been offered for the wounded, accommodation made for Belgian refugees, and provision for the comfort and recreation of our soldiers in training. The latest suggestion is on a larger scale than any that has hitherto been made, and presents some novel features. After consultation with Sir Walter Lawrence and other representatives of the War Office, the Guardians of Brighton Parish have decided to place at the disposal of the Government the whole of their workhouse and infirmaries for the treatment of the wounded and sick amongst our Indian troops engaged in the war. From 1,500 to 2,000 beds will thus be rendered available. It is proposed to transfer the whole of the institution to the War Office, which will be responsible for its administration. The buildings, which include a modern and well-equipped hospital, are admirably situated on Race Hill, and possess four acres of garden land, in addition to an acre and a-half not under cultivation.

The problem of dealing with our Indian citizens so as not to offend their caste system and their ritual and racial customs would be an insuperable difficulty with a staff ignorant of them and of their importance. Poor-Law Guardians and their officers could not be expected to cope successfully with these difficulties. That is no doubt the reason which has induced the War Office to stipulate and the Brighton Guardians to agree that the administration shall be entirely in the hands of the former body. It was essential that the War Office should have a large, suitably placed institution, ready for immediate occupation, at their disposal. For the time being, therefore, the Brighton Workhouse will cease to be a Poor-Law institution, and will become a purely military hospital.

The patriotism of the Brighton Board of Guardians is worthy of praise, for it is no small matter for a Board of Guardians to give up practically all its institutions. It means the abandonment of an extremely interesting part of its functions, and one in which its members naturally feel a great pride. It means hard work in finding fresh suitable accommodation for the indoor poor and sick. Provision will be made for some of the inmates in the Warren Farm Schools, but many of them will have to be boarded out in the buildings of neighbouring Boards of Guardians, and the system of outdoor relief will have to be extended. The position of the existing staff will no doubt receive careful and sympathetic consideration. The services of the medical and nursing staff will probably be retained by the War Office. With the assistance of officers acquainted with the language and customs of their new patients the retention of the members of these staffs will present few difficulties and obvious advantages. There is no reason, also, why most of the clerical, domestic, and steward's staff should not also be kept, and the residue will probably be required at the Warren Farm Schools and the other institutions to which the displaced inmates will be sent. It should be easy, therefore, to make the transfer without inflicting any hardship upon the officials.

The matter is of importance because of what has occurred in similar cases under other Boards of Guardians. The Oxford Guardians, for instance, recently transferred their workhouse to the War Office for use as a military hospital. In doing so the Guardians dismissed the whole of their staff, with the exception of the master, matron, medical officer, and chaplain, paying them three months' salaries and the value of their emoluments for this time. This is a grave injustice to officials who have always regarded their appointments as permanent, terminable only by voluntary resignation or as the result of grave misconduct. It is not right, and it is not necessary that they should suffer because Poor-Law institutions are required for the wounded. The ordinary work of the Poor Law must still go on, and it should be practicable in most cases to arrange for the temporary transfer of officials to those authorities with whom arrangements are made for the reception of displaced inmates.

